# The characterization of a novel monoclonal antibody against CD93 unveils a new antiangiogenic target

**DOI:** 10.18632/oncotarget.1887

**Published:** 2014-04-07

**Authors:** Maurizio Orlandini, Federico Galvagni, Monia Bardelli, Marina Rocchigiani, Claudia Lentucci, Francesca Anselmi, Alessio Zippo, Luca Bini, Salvatore Oliviero

**Affiliations:** ^1^ Department of Biotechnology, Chemistry and Pharmacy, University of Siena, Via A. Moro, 2 – 53100 Siena, Italy; ^2^ Department of Life Sciences, University of Siena, Via A. Moro, 2 – 53100 Siena, Italy; ^3^ HuGeF, Via Nizza, 52 – 10126 Torino, Italy; ^4^ Department of Life Sciences and Systems Biology, University of Torino, Via Accademia Albertina, 13 – 10123 Torino, Italy; ^5^ Novartis Vaccines, Via Fiorentina, 1 – 53100 Siena, Italy; ^6^ INGM, Via F. Sforza, 35 – 20122 Milano, Italy

**Keywords:** monoclonal antibody, endothelial cell, angiogenesis, C1qRp, AA4

## Abstract

The inhibition of tumor angiogenesis is one of the main challenges in cancer therapy. With the aim of developing monoclonal antibodies able to inhibit angiogenesis, we immunized mice with proliferating human umbilical vein endothelial cells. We generated a library of monoclonal antibodies able to recognize antigens expressed on endothelial cells and screened the antibodies for their ability to inhibit endothelial cell proliferation, migration, and sprouting *in vitro*. Here, we show that the antibody, designated as 4E1, is able to neutralize the formation of new vessels both *in vitro* and *in vivo* without affecting endothelial cell survival. By mass spectrometry we identified CD93 as the antigen bound by 4E1 and mapped the recognized epitope. CD93 is a transmembrane protein heavily glycosylated preferentially expressed in the vascular endothelium. CD93 silencing by lentiviral-mediated small hairpin RNA expression impairs human endothelial cell proliferation, migration, and sprouting. Altogether these findings reveal 4E1 as a novel antiangiogenic antibody and identify CD93 as a new target suitable for antiangiogenic therapy.

## INTRODUCTION

Angiogenesis supplies all cellular tissues with oxygen and nutrients and plays a key role not only during embryo development and physiologic processes but also in several diseases, of which cancer is perhaps the most serious [1, 2]. Since without blood vessels tumors cannot grow beyond a critical size or metastasize to distant organs, it appeared immediately evident that suppression of angiogenesis could be a valid strategy for the development of anticancer treatments. Thus, in recent years angiogenesis inhibitors have been developed and approved for a wide range of cancer types [3]. Among them, monoclonal antibodies (mAbs) revealed useful tools for cancer treatment [3, 4]. Bevacizumab (Avastin, Genentech/Roche), a function-blocking antibody to VEGF, was the first to show clinical benefit in patients with colorectal cancer when combined with chemotherapy [5]. However, the use of this drug combination highlighted that antiangiogenic therapy is hampered by reactive resistance-response of cancer cells and Bevacizumab can function by either blocking this resistance-response or by normalizing tumor angiogenesis allowing a better drug delivering to tumor cells [6]. Thus, despite the use of antiangiogenic strategies has proved successful for some patients, antiangiogenic therapy still needs to improve efficacy, avoid resistance, and minimize toxicity [7-9]. Antigens involved in angiogenesis that are suitable for antibody-based therapeutics are usually proteins that support the formation of new vasculature, such as the growth factors belonging to the VEGF family [10, 11]. However, the discovery of new antigens with complementary functions, could offer novel opportunities for improved treatment.

In the present study, exponentially growing endothelial cells were used to generate a novel murine IgG1 antibody, 4E1, which revealed able to inhibit angiogenesis both *in vitro* and *in vivo* experiments. Biochemical analyses showed that CD93 is the antigen recognized by 4E1 and deletion mutant analyses mapped the binding site of 4E1 on CD93 extracellular domain.

Human CD93 is a cell surface glycoprotein composed of 652 amino acids. The predicted molecular mass of CD93 is 68 kDa, but its relative migration in SDS-PAGE under reducing conditions is 126 kDa due to a high degree of glycosylation and the presence of regions with high contents of proline and charged amino acids [12-14]. The structural domain analysis within CD93 from N- to C-terminus reveals the presence of a C-type lectin-like domain (CTLD), five epidermal growth factor (EGF)-like repeats, a mucin-like domain, a transmembrane domain, and a cytoplasmic domain [12]. Accordingly to Wu and colleagues [15], these domains were respectively designated as D1, D2, D3, D4, and D5, while we designated DX a 79-amino acid domain of unknown structural function localized between D1 and D2 domains.

Many evidences suggest that CD93 may play a role in the endothelium. Although human CD93 is expressed in different cellular types, its predominant site of expression is the vascular endothelium [16-18]. The mouse homologue of CD93, AA4, is expressed on vascular endothelial cells in the developing embryo, particularly during the remodeling of blood vessels, consistent with a role for CD93 in angiogenesis [19]. Moreover, the surface protein CD93 is susceptible to protein ectodomain cleavage, or shedding, that may contribute to the angiogenic process [20]. Indeed, recently it has been reported that the soluble EGF-like domain of CD93 is a novel angiogenic factor [15]. However, despite these observations the molecular function of CD93 in angiogenesis must still be clarified.

Here, using an anti-CD93 monoclonal antibody and inhibiting the function of CD93 in human endothelial cells, we demonstrate the involvement of CD93 in the control of endothelial cell function and identified a potential new target for antiangiogenic treatment of diseases.

## RESULTS

### The mAb 4E1 inhibits *in vitro* and *in vivo* angiogenesis

**Figure 1:**
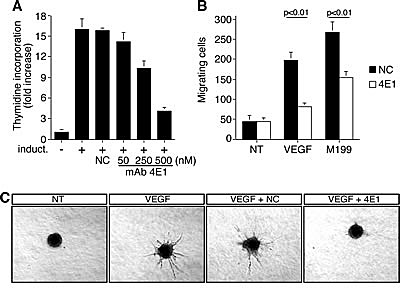
The mAb 4E1 affects cell proliferation, migration, and in vitro sprouting of human endothelial cells A: Cell proliferation expressed as thymidine uptake in HUVEC. Cells were grown in 96-well-plates, serum starved, and induced to proliferate with complete medium (induct.) in the presence of different concentrations of 4E1 or unrelated purified antibodies (NC, 500 nM). B: Migration assay on HUVEC in the presence of 4E1 (500 nM) or unrelated purified antibodies (NC, 500 nM). Cells were grown in growth factor-depleted culture medium and plated in Boyden chambers. Chemotaxis was stimulated with 10 ng/ml VEGF (VEGF) or complete medium (M199). Migratory cells were stained and counted under a light microscope. C: Sprouting of HUVEC spheroids embedded into collagen gels in the absence (NT) or presence of 10 ng/ml VEGF (VEGF). NC, unrelated purified antibodies (500 nM), mAb 4E1 (500 nM). A representative experiment is shown (original magnification, x40). Data represent the ± SD of three-five independent experiments each in triplicate.

Angiogenesis involves both proliferation and migration of capillary endothelial cells. Since cycling endothelial cells express a different antigen profile compared to quiescent cells found in stable vessels [21, 22], we immunized mice with proliferating HUVEC to raise mAbs able to block the function of proteins involved in the angiogenic process. First, we screened antibodies able to selectively recognized antigens on the surface of endothelial cells by flow cytometry (Supplemental Fig. 1). Then, we purified these mAbs and used them to challenge the main traits of the angiogenic process: proliferation, migration, and differentiation. We selected the mAb 4E1 (isotype IgG1, k chain) that revealed to be competent for the inhibition of HUVEC proliferation in a dose-dependent manner, whereas a unrelated antibody did not influence cell proliferation even at high concentrations (Fig. 1A). The analysis of cell migration by using the Boyden chamber assay showed a significant reduction of endothelial cell migration after stimulation with growth factors in the presence of 4E1 compared to control cells (Fig. 1B). Furthermore, the ability of HUVEC to sprout up from spheroids embedded into collagen gels following VEGF stimulation was inhibited almost completely when the spheroids were incubated with 4E1, whereas an unrelated antibody did not affect significantly sprout number and length (Fig. 1C), indicating that the mAb 4E1 exerts an antiangiogenic effect.

**Table:** Table I: Protein identification by mass spectrometry Proteins corresponding to the bands isolated from a gel are indicated. AN, accession number retrieved by UniProt knowledgebase (http://www.uniprot.org/). The number of measured peptide masses matching the protein, the percentage of the protein sequence covered by the matching peptides (Seq. cov. %), and the probabilistic score are shown. Scores greater than 70 are usually considered significant matches. Theor. Mw, theoretical molecular weight calculated by Compute pI/Mw tool (http://web.expasy.org/compute_pi/)

Band	AN	Protein	Peptides	Seq. cov. %	Score	Theor. Mw (Da)
1	Q9NPY3	Complement component C1q receptor	13	26	125	66451
2	Q9NPY3	Complement component C1q receptor	12	25	118	66451
3	Q9NPY3	Complement component C1q receptor	13	27	126	66451

We further investigated the ability of HUVEC to form capillary-like structures when cultured on Matrigel, which is a process mimicking tube formation during angiogenesis *in vivo*. When HUVEC were grown on Matrigel in the presence of 4E1, tubulogenesis was strongly impaired with respect to cells grown in the presence of an unrelated antibody, since few and broken tubes were produced (Fig. 2A and B). Moreover, to assess the antiangiogenic effect of 4E1 *in vivo*, we performed a Matrigel plug assay in mice. Exponentially growing HUVEC were included in Matrigel in the presence of 4E1 or an unrelated antibody and injected into nude mice. Matrigel plugs were excised and processed for immunofluorescence staining (Supplemental Fig. 2). Microvessel density and vessel length were determined as described in Materials and methods. Importantly, microvessel density resulted decreased in plugs containing the mAb 4E1 (Fig. 2D) and HUVEC within the plugs containing 4E1 significantly decreased their ability to form the tubular structures observed in control plugs (Fig. 2C and E). Collectively, our data show that the mAb 4E1 inhibits the formation of new vessels by preventing endothelial cell proliferation, migration, and formation of capillary-like structures both *in vitro* and *in vivo* assays.

**Fugure 2:**
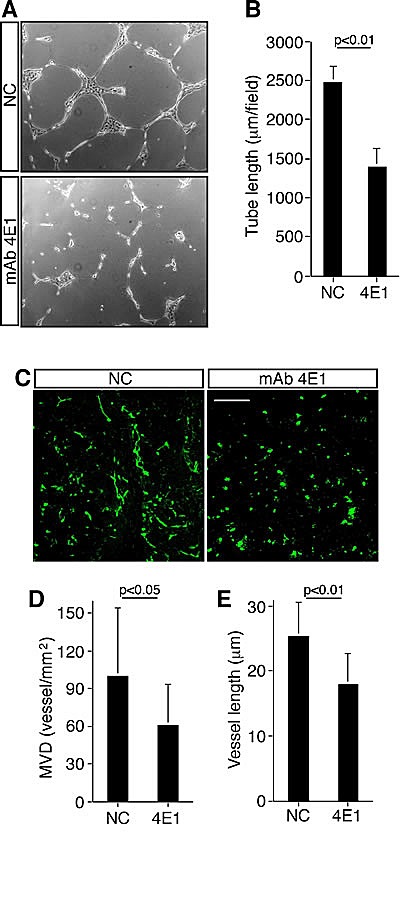
The mAb 4E1 impairs angiogenesis in vivo A: HUVEC were grown on Matrigel in the presence of 4E1 (500 nM) or unrelated antibodies (NC, 500 nM) and the formation of capillary-like structures was observed 20 h after seeding. A representative experiment is shown (original magnification, x100). B: Quantification of tube length was performed based on the results shown in panel A. Results were expressed as means ± SD of four different fields randomly chosen from each group from three independent experiments. C: Nude mice were injected subcutaneously with Matrigel containing HUVEC in the presence of 4E1 or unrelated antibodies (NC). Matrigel plugs were excised, sectioned and subjected to immunofluorescence analysis. Representative sections, stained with anti-von Willebrand factor antibodies, are shown. Scale bar represents 150 μm. D: Quantification of microvessel density (MVD) was performed based on the results shown in panel C as reported in Materials and methods. E: Quantification of vessel length was performed based on the results shown in panel C as reported in Materials and methods. At least ten sections/plug were examined. Data represent the ± SD of three independent experiments.

### CD93 is the target recognized by the mAb 4E1

**Figure 3:**
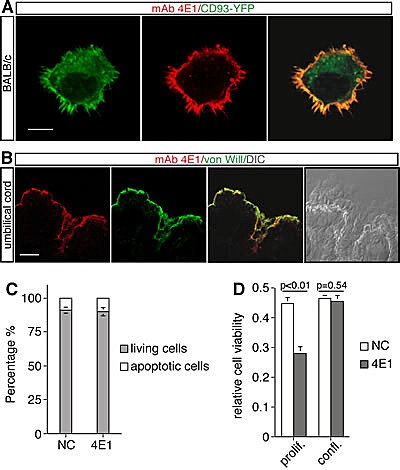
The mAb 4E1 recognizes the membrane protein CD93 and does not affect endothelial cell survival A: Mouse BALB/c fibroblasts were transfected with a construct expressing the human chimeric protein CD93-YFP under the control of a constitutive promoter. Cells were plated on glass coverslips, fixed and subjected to immunofluorescence analysis in the absence of permeabilization by using the mAb 4E1. Scale bar represents 8 μm. B: Vein section through human umbilical cord stained by immunofluorescence using anti-von Willebrand factor (von Will) and 4E1 antibodies. The merge of the double staining and a DIC image of the same cross section are shown. Scale bar, 22 μm. C: The effect of 4E1 treatment on survival of growth-arrested endothelial cells. HUVEC were grown to confluence and treated with 500 nM of 4E1 or unrelated antibodies (NC) for 48 h. The apoptotic percentage was calculated by flow cytometric analysis of nuclei stained with propidium iodide. D: Cell viability assay on proliferating (prolif.) and quiescent (confl.) endothelial cells treated with 500 nM 4E1 or unrelated antibodies (NC) for 96 h. The optical density values were expressed as relative cell viability. All data represent the ± SD of three independent experiments.

To unveil the protein recognized by the mAb 4E1, cell extracts from proliferating HUVEC were immunoprecipitated with 4E1 and the electrophoretic bands were excised and analyzed by mass spectrometry. Three isolated bands corresponded to CD93 also known as the complement component C1q receptor (C1qRp) (Table I), a type-I transmembrane glycoprotein highly expressed on the endothelium. To confirm the binding of 4E1 on CD93, we cloned CD93 and expressed the protein in mouse BALB/c fibroblasts, which do not express endogenous CD93 (Supplemental Fig. 3). Immunofluorescence analysis of BALB/c fibroblasts transfected with a CD93-YFP plasmid and stained without permeabilization by using 4E1 revealed that the CD93-YFP chimeric protein is detectable both at the cell surface and in the cytoplasm whereas 4E1 clearly colocalizes with CD93-YFP only to the cellular periphery as shown by the merged image (Fig. 3A), showing that 4E1 recognizes CD93.

Since we developed mAbs by immunizing mice with exponentially growing HUVEC, we asked whether CD93 was preferentially expressed in proliferating endothelial cells. To address this issue, HUVEC were plated at low density, left to grow and maintained at confluence for one week. Proteins were extracted at different time-points of cell growth and subjected to Western blot analysis. Comparison of the protein levels revealed that there are no differences in the pattern of expression of CD93 both in proliferating and resting cells and that CD93 is highly expressed in endothelial cells independently of cell growth conditions (Supplemental Fig. 4). Accordingly, immunofluorescence analysis of umbilical vein sections showed that 4E1 staining is localized in endothelial cells that lined the interior surface of the cord vessels (Fig. 3B), indicating that CD93 is expressed in quiescent endothelial cells of healthy blood vessels. For this reason, we asked whether the antiangiogenic activity of 4E1 could also exert a cytotoxic effect on resting endothelial cells. To address this question, HUVEC were grown at confluence for few days in the presence of 4E1 or unrelated antibodies and subjected to cell survival and viability assays. Flow cytometric analysis with propidium iodide staining showed that, in cell-cycle arrested endothelial cells (Supplemental Fig. 5), the treatment for 48 h with 4E1 at the higher concentration used for all experiments, does not change the percentage of apoptotic cells with respect to control cells (Fig. 3C). Moreover, the prolonged treatment of endothelial cells with high concentrations of 4E1 impaired cell viability of proliferating cells, whereas the same treatment did not alter cell viability of quiescent cells with respect to the control (Fig. 3D). Taken together, our results show that the mAb 4E1 binds CD93 and exerts its antiangiogenic activity without affecting survival of resting endothelial cells.

### Mapping of the 4E1 epitope binding site

**Figure 4:**
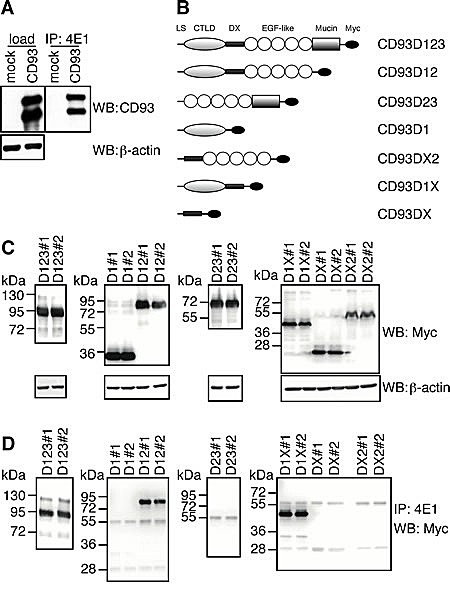
4E1 recognizes a conformational epitope localized on a region overlapping D1 and DX domains of CD93 A: Human Lenti-X 293T cells were transiently transfected with empty vector (mock) or a construct expressing human CD93 full-length. Cell extracts were immunoprecipitated with 4E1 and immunoprecipitates were analyzed by Western blotting with anti-CD93 antibodies (H190). Equal loading of cell lysates was confirmed by using anti-β-actin antibodies. B: The schematic diagram illustrates the deletion mutants of the CD93 extracellular domains fused with a 6X Myc tag. The deletion mutants were designated in accordance with Wu and colleagues [15]. LS, signal peptide; CTLD, C-type lectin-like domain; X, domain of unknown structural function; EGF-like, EGF-like repeats; Mucin mucin-like domain; Myc, 6X Myc tag. C: Lenti-X 293T cells were transiently transfected to generate recombinant CD93 domain proteins fused with a 6X Myc tag. For each different deletion mutant two different cDNA constructs were transfected (#1 and #2). Cell extracts were analyzed by immunoblotting using anti-Myc antibodies. Anti-β-actin antibodies were used to confirm equal loading. D: Cell lysates from cells transfected as in C, were subjected to immunoprecipitation analyses by using 4E1. Immunoprecipitates were analyzed by Western blotting with anti-Myc antibodies.

**Figure 5:**
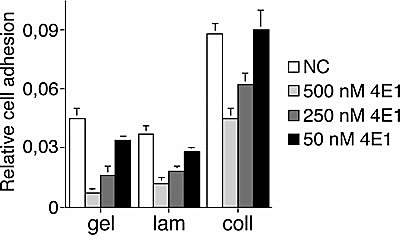
The mAb 4E1 impairs endothelial cell adhesion on different substrates Gelatin (gel), laminin (lam) and collagen (coll) adhesive substrates were used for coating. HUVEC, grown in complete medium, were biochemically detached, incubated in the presence of different concentrations of 4E1 (50, 250, and 500 nM) or unrelated antibodies (500 nM, NC) for 15 min in ice and allowed to adhere on substrate-coated surfaces for 10 min. Fixed cells were stained with crystal violet solution, and the optical density values were expressed as relative cell adhesion. Data represent the ± SD of four independent experiments each in triplicate.

Since it has been reported that the EGF-like domain of CD93 is a potent angiogenic factor, which exerts its activity through the binding to EGFR and the activation of downstream ERK phosphorylation [15], we asked whether 4E1 could interfere with this interaction. To address this issue, we proceeded with the identification of the binding site of 4E1 on CD93. First, Lenti-X 293T cells were transfected with CD93 full-length and the cell lysates were analyzed by Western blotting and immunoprecipitation assays. Whereas the mAb 4E1 did not show any binding to CD93 by immunoblotting analysis under denaturing conditions (data not shown), immunoprecipitation with 4E1 and immunoblotting with anti-CD93 antibodies revealed that 4E1 is able to bind the whole molecule of CD93 from non-denatured cell extracts (Fig. 4A), clearly indicating that 4E1 recognizes a conformational epitope. Since the structure of human CD93 consists of distinct domains [23], we extended our mapping analysis generating CD93 deletion mutants and testing the ability of 4E1 to immunoprecipitate these recombinant proteins (Fig. 4B). Each chimeric mutant was fused with the Myc epitope tag and two different constructs were transfected in Lenti-X 293T cells, which do not express wild-type CD93 (Fig. 4A). Then, mutant expression was checked by Western blotting analysis with anti-Myc antibodies (Fig. 4C). Immunoprecipitation analyses of the deletion constructs allowed us restricting the region of CD93 containing the epitope recognized by 4E1 to a region overlapping the domains D1 and DX, with both extracellular domains necessary to fold the epitope correctly (Fig. 4D). Accordingly, we observed that 4E1 does not interfere with the CD93-dependent activation of ERK1/2 (Supplemental Fig. 6), which has been previously shown to be dependent on the EGF-like domain of CD93 [15]. Instead, in agreement with a possible involvement of CD93 in endothelial cell adhesion and migration [16, 19], we observed that 4E1 is able to inhibit endothelial cell attachment to different adhesive substrates in a dose-dependent manner (Fig. 5), suggesting that inhibition of cell adhesion is the inhibitory effect exerted on endothelial cells by 4E1.

### CD93 knockdown impairs endothelial cell function

**Figure 6:**
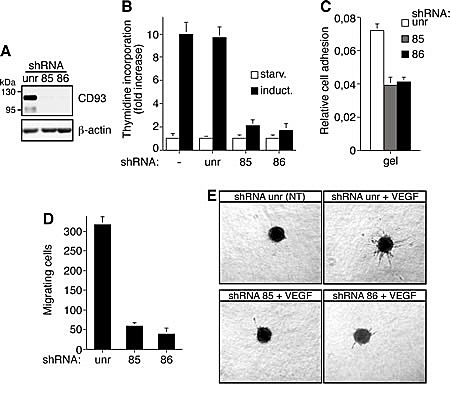
CD93 silencing affects cell proliferation, adhesion, migration, and in vitro sprouting of human endothelial cells HUVEC were infected with a lentiviral vector expressing unrelated (unr) or CD93 shRNAs (clone 85 or 86). A: Cell extracts from shRNA expressing HUVEC were analyzed by Western blotting using anti-CD93 (H190) and anti-β-actin antibodies to confirm equal loading. B: Cell proliferation expressed as thymidine uptake in infected HUVEC. Cells were grown in 96-well-plates, serum starved (starv) and induced with complete medium (induct). Not infected endothelial cells were also analyzed (-). C: Cell adhesion assay of infected HUVEC. Cells were biochemically detached and allowed to adhere on gelatin-coated surfaces for 15 min. Fixed cells were stained with crystal violet solution and the optical density values were expressed as relative cell adhesion. D: Migration assay on infected HUVEC. Cells were grown in growth factor-depleted culture medium and plated in Boyden chambers. Chemotaxis was stimulated with 10 ng/ml VEGF. Migratory cells were stained and counted under a light microscope. E: Sprouting assay of infected endothelial cells embedded into collagen gels in the absence (NT) or presence of 10 ng/ml VEGF (VEGF). A representative experiment is shown (original magnification, x40). Data represent the ± SD of four-five independent experiments each in triplicate.

To further investigate the involvement of CD93 in the regulation of the angiogenic process, we silenced CD93 in HUVEC by two lentiviral constructs expressing two independent CD93 shRNAs (clones 85 and 86). HUVEC infected with lentiviruses expressing either CD93 shRNA showed reduced CD93 protein levels while no CD93 reduction was revealed upon infection with a lentivirus expressing an unrelated shRNA (Fig. 6A). Since in response to proangiogenic stimuli quiescent endothelial cells become activated and involved in proliferation, migration, and differentiation, we assessed the effect of CD93 silencing in growth factor-stimulated endothelial cells. First, we observed that DNA synthesis, determined by thymidine uptake, is strongly decreased in growth factor-induced endothelial cells expressing CD93 shRNAs with respect to control cells (Fig. 6B). Furthermore, cell adhesion and VEGF-stimulated migration of endothelial cells silenced for CD93 resulted significantly decreased compared to control cells expressing an unrelated shRNA (Fig. 6C and 6D). Finally, the ability of HUVEC to form VEGF-induced sprouts from spheroids embedded into collagen gels was nearly totally inhibited when endothelial cells were transduced with lentiviruses carrying CD93 shRNAs, while sprouting was not impaired in HUVEC infected with lentiviruses carrying the control shRNA (Fig. 6E). Taken together these results indicate that CD93 plays a role in endothelial cell proliferation and migration to control the organization of endothelial cells into vessel structures.

**Table:** Table II: Deletion mutants of CD93 extracellular domains The recombinant domain proteins of CD93 cover the amino acid sequence reported (a.a.). The protein sequence of human CD93 is numbered according to the sequence with GenBank™ accession number AAH28075.1. cDNA fragments were PCR-amplified by using the forward and reverse primers indicated (oligos)

CD93 domains	a.a.	oligos
CD93D1X23	M1-K580	P389 GAGAggatccGCCGCCACCGGGATGG for P390 GAGAggatccCTTTTGCCCGTCAGTGC rev
CD93D1X2	M1-G470	P389 for P394 GAGAggatccCCCCATGGTGCAAGAGAC rev
CD93D23	V258-K580	P397 GAGAggatccGTCAGCCCCAAGTATGG for P390 rev
CD93D1	M1-G177	P389 for P393 GAGAggatccGCCCTCAATGTTACTTCC rev
CD93DX2	F178-G470	P434 GAGAggatccTTCGTGTGCAAGTTCAGC for P394 rev
CD93D1X	M1-C257	P389 for P433 GAGAggatccACAGAGGGGGCCCGAG rev
CD93DX	F178-C257	P434 for P433 rev

## DISCUSSION

In this report, we characterize a novel antiangiogenic antibody, designated as 4E1. We showed that treatment with 4E1 impairs proliferation, migration, and sprouting of human endothelial cells. Murine angiogenesis assays confirmed that 4E1 is able to halt angiogenesis *in vivo*, demonstrating that 4E1 neutralizes an antigen exposed on endothelial cells that is involved in the regulation of the angiogenic process. By mass spectrometry we identified this antigen as CD93.

Our study supports a model positing that CD93 is involved in the regulation of endothelial cell function during angiogenesis. Indeed, by RNA interference we showed that in endothelial cells CD93 knockdown strongly affects proliferation, migration, and formation of capillary-like structures. CD93 is an adhesion molecule involved in biological processes that require dynamic cytoskeletal rearrangement such as migration [24]. Moreover, the linkage of CD93 to actin by Moesin contributes to a redistribution of the cytoskeleton that has been shown to be essential for migration and adhesion [23]. Consistent with these observations, we observed that CD93 has a pattern of localization similar to Moesin, being mainly concentrated in the apical domain of adhering endothelial cells (data not shown) [25], 4E1 is able to inhibit endothelial cell adhesion on different substrates, and CD93 silencing impairs cell attachment to the substrate, suggesting a role for CD93 in adhesion of endothelial cells.

Although CD93 is predominantly expressed in the endothelium of mouse and human origin [16, 19], CD93-deficient mice are viable and show no gross abnormalities in their vascular development [26]. This discrepancy could be explained by gene compensations in the knockout animals, as observed with other genes [27], because CD93 belongs to a family of transmembrane glycoproteins, which also includes endosialin and thrombomodulin [24].

It has been reported that soluble CD93 fragments are released during inflammation and their presence in the plasma of patients is a biomarker for coronary artery disease [28, 29]. Soluble CD93 may be the product of ectodomain cleavage of membrane CD93 mediated by matrix metalloproteinases, known to be key factors in vascular remodeling [20]. Moreover, the EGF-like domain of soluble CD93 has recently been shown to exhibit proangiogenic effects on the endothelium acting as an EGFR agonist [15]. In agreement with our mapping studies, which localized the 4E1 epitope on CD93 outside the EGF-like domain, we found that 4E1 does not impair CD93-dependent EGFR activation. Taken together, these results suggest that CD93 plays different angiogenic functions both as membrane-intercalated protein or soluble fragments and 4E1 can discriminate between them.

As selection for resistance is a hallmark of an effective antiangiogenic therapy and a consequence of tumor regression, for improving the treatment of human cancer with angiogenesis inhibitors, a promising strategy involves multi-targeting of parallel proangiogenic signaling pathways to circumvent evasive mechanisms [7, 8]. Thus, the neutralization of CD93 in combination with other drugs or blocking agents could be revealed instrumental in antiangiogenic treatment of cancer. Importantly, endothelial cell survival and viability of quiescent cells were not affected by 4E1 treatment. Although 4E1 was raised in mice using exponentially growing HUVEC, we found that CD93 is expressed at the same levels both in growing and quiescent cells but 4E1 is able to specifically affect only proliferating cells, suggesting that CD93 plays its angiogenic function when endothelial cells shuttle from a quiescent to a proliferating state. Consistent with these results, in matrigel plug assays microvessel density, in which isolated cells were considered as a vessel, resulted slightly decreased indicating that 4E1 targeting CD93 inhibits branching of new vessels but not survival of endothelial cells localized in the plug. In conclusion, our results suggest that CD93 represents a new antigen on endothelial cells, whose targeting may be an useful tool to halt angiogenesis.

## MATERIALS AND METHODS

### Cell culture and transfections

HUVEC were isolated from umbilical cords collected from uncomplicated pregnancies of consenting healthy patients. For each experiment at least three independent extractions of HUVEC were used. Cells were cultured on gelatin-coated Petri dishes in 1:1 (v/v) M199 (containing 20% fetal bovine serum and 100 μg/mL brain extract) and EBM-2 with supplements (Lonza, Basel, Switzerland) culture media and used in passages 3-6. Mouse BALB/c (ATCC, Manassas, VA, USA) and human Lenti-X 293T (Clontech Lab, Mountain View, CA, USA) cell lines were cultured using standard conditions. Transient transfection experiments were performed by using PolyFect reagent (Qiagen, Hilden, Germany) in BALB/c cells and lipofectamine 2000 (Invitrogen, San Diego, CA, USA) in Lenti-X 293T cells according to instructions from the manufacturers.

### Monoclonal antibody production

mAbs were generated as previously described [30]. Briefly, female BALB/c mice were fourfold injected with 8 × 10^6^ cultured HUVEC released from the substrate by using detaching buffer (10 mM EDTA in PBS) and resuspended as single cells in PBS. Immune spleen cells were fused with X63-Ag8 myeloma cells. 1021 hybridomas were screened for their ability to produce mAbs that bind endothelial cell surface antigens by flow cytometry. Briefly, after biochemical detachment, HUVEC were incubated in PBS containing 5% fetal bovine serum to block unspecific binding and labeled with primary antibodies diluted in PBS containing 1% bovine serum albumin. Staining was revealed using anti-mouse conjugated secondary antibodies. Then, positive mAbs were selected for their ability to not recognize antigens expressed on the surface of human epithelial HEK-293 cells (ATCC). Data acquisition and analysis were performed in a Becton Dickinson FACS Calibur flow cytometer using CellQuest software. 61 different antibodies that recognized endothelial surface molecules were purified by affinity chromatography with HiTrap Protein G columns (GE Healthcare, Piscataway, NJ, USA) and used in inhibition assays.

### Cell proliferation, cell viability, apoptosis, migration, adhesion, and *in vitro* angiogenesis assays

Cell proliferation was evaluated by thymidine incorporation as previously described [30]. Cell viability was quantified by spectrophotometric measurement of the mitochondrial dehydrogenase activity by using the CellTiter 96^®^ AQ_ueous_ one solution cell proliferation kit (Promega, Fitchburg, WI, USA) according to manufacturer instructions. The optical density values were measured at 490 nm using a microtiter plate reader (VERSAmax™, Molecular Devices, Sunnyvale, CA, USA). The percentage of apoptotic nuclei was assessed as previously described by propidium iodide staining followed by flow cytometric analysis [31]. For adhesion assays, the following adhesive substrates were dissolved in PBS and used for coating of 96-well plates (2h at 37° C): rat collagen (mainly type I collagen, 10 μg/mL) from Roche Diagnostics (Mannheim, Germany), porcine gelatin (1%) and mouse laminin (10 g/mL) from Sigma-Aldrich. Nonspecific adhesion was blocked with M199 culture medium containing 3% bovine serum albumin. 2.5 × 10^4^ HUVEC, grown in complete medium, were biochemically detached, and allowed to adhere on substrate-coated surfaces. 10 min after plating, cells were fixed in 3% paraformaldehyde, stained with 0.05% (w/v) crystal violet solution, and the optical density values were measured at 570 nm. Chemotaxis analysis with Boyden transwell chambers, endothelial cell sprouting assay in collagen gels and formation of capillary-like tube structures in Matrigel were performed as previously described [32, 33]. For neutralization assays, the mAb 4E1 was used at different concentrations based on available dosing schedules previously used for other antiangiogenic antibodies targeting surface proteins not belonging to the VEGF family [34, 35].

### Matrigel plug assay

Female 10-week-old nude mice were obtained from Charles River (Wilmington, MA, USA). The mice were subcutaneously injected in the dorsal region with 500 μL of growth factor reduced Matrigel (BD Biosciences, San Jose, CA, USA) mixed 2:1 (v/v) with HUVEC (6 × 10^6^ cells/mL). For assays with neutralizing antibody, the mAb 4E1 was incorporated into the Matrigel. Matrigel plugs were surgically removed after 2 weeks, embedded in OCT, cryostat sectioned and processed for immunofluorescence analysis. For evaluation of microvessel density, areas with the highest microvessel concentration were visually identified and several histological fields were chosen for the measurement. The area of the histological fields was 533,448 μm^2^ (20x objective lens and 10x ocular lens). Isolated cells or clusters of cells displaying a strong fluorescent staining were considered as a vessel. Quantitative analysis of digital images was performed using ImageJ software. The number of von Willebrand factor-positive structures was counted in a minimum of five fields/section. The mean vessel length/field was calculated in ten fields/section. Statistical significance of the data was evaluated using an unpaired Student's t-test.

### Immunofluorescence microscopy

For histological analysis, C57BL/6 mice were sacrificed and portions of tail were dissected and frozen in liquid nitrogen. Portions of human umbilical cords were dissected and frozen in liquid nitrogen. 10 μm cryostat sections from tails, umbilical cords or Matrigel plugs were fixed in 3% paraformaldehyde for 15 min at room temperature. Cells were seeded onto gelatin-coated glass coverslips and fixed in 3% paraformaldehyde. Histological sections and cells were treated as previously described [33, 36]. The following primary antibodies were used: mouse anti-CD93 (mAb 4E1, 0.6 mg/mL) 1:25; rabbit anti-von Willebrand factor (Dako, Glostrup, Denmark); rat anti-CD31 (BD Pharmingen, San Diego, CA, USA). Fluorescent images were captured using a Leica TCS SP2 laser-scanning confocal microscope.

### Cloning and plasmid constructs

The full-length cDNA of human *CD93* (GenBank™ accession number NM_012072.3) was amplified using the BcaBEST™ RNA PCR kit (Takara Bio Inc., Otsu, Japan) from reverse transcription of total RNA extracted from HUVEC (oligonucleotides CD93F, 5'-GAGAGGATCCGCCGCCACCGGGATGGC CACCT-3' and CD93R, 5'-GAGAGAGCGGCCGCCTCTAG GGCCACCTCAC-3'. The PCR fragments were cloned in pcDNA3 cloning vector (Invitrogen). *CD93* full-length cDNA was subcloned into pEYFP-N1 vector (Clontech Lab), positioning the fluorescence tag at C-terminal. To generate the chimeric constructs containing the extracellular domains of CD93 fused to Myc tag, DNA fragments were PCR-amplified from a cDNA clone corresponding to the complete sequence of the human *CD93* gene and cloned in pCS2^+^myc tag vector [37]. The chimeric constructs and the primers used for DNA amplification are reported in Table II. All constructs were confirmed by sequencing.

### Mass spectrometry

Protein identification was carried out by peptide mass fingerprinting on an Ultraflex III MALDI-TOF/TOF mass spectrometer (Bruker Daltonics, Billerica, MA), as previously described [38].

### RNA interference-mediated knockdown of CD93

Silencing experiments were performed using retroviral vectors pLKO.1 from the TRC lentiviral shRNA library (Open Biosystems, Huntsville, AL, USA) expressing specific shRNAs for human CD93 (oligonucleotide TRCN0000029085, designated as 85; and oligonucleotide TRCN0000029086, designated as 86). Recombinant lentiviruses were produced and used for infection experiments as previously described [33].

### Immunoprecipitation and immunoblotting analyses

Immunoprecipitation and immunoblotting analyses were performed as previously described [33]. The following primary antibodies were used for immunoblotting: rabbit anti-CD93 (H190) and mouse anti-c-Myc (9E10, Santa Cruz Biotechnology, Santa Cruz, CA, USA); mouse anti-β-actin (Sigma-Aldrich, St Louis, MO, USA); anti-p44/42 MAPK and anti-phospho-p44/42 MAPK (Cell Signaling, Danvers, MA, USA). For immunoprecipitations, the protein extracts were incubated for 2 h at 4° C with the mAb 4E1 coupled to Dynabeads® Pan Mouse IgG (Invitrogen).

## SUPPLEMENTARY FIGURES


