# Supplementary material for: The characterization of a novel monoclonal antibody against CD93 unveils a new antiangiogenic target
Source: Oncotarget. 2014 Apr 7;5(9):2750–60. doi: 10.18632/oncotarget.1887 (PMC4058042; doi:10.18632/oncotarget.1887)
Supplement: Supplementary file 1 [file oncotarget-05-2750-s001.pdf]

The characterization of a novel monoclonal antibody against CD93 unveils a new antiangiogenic target.

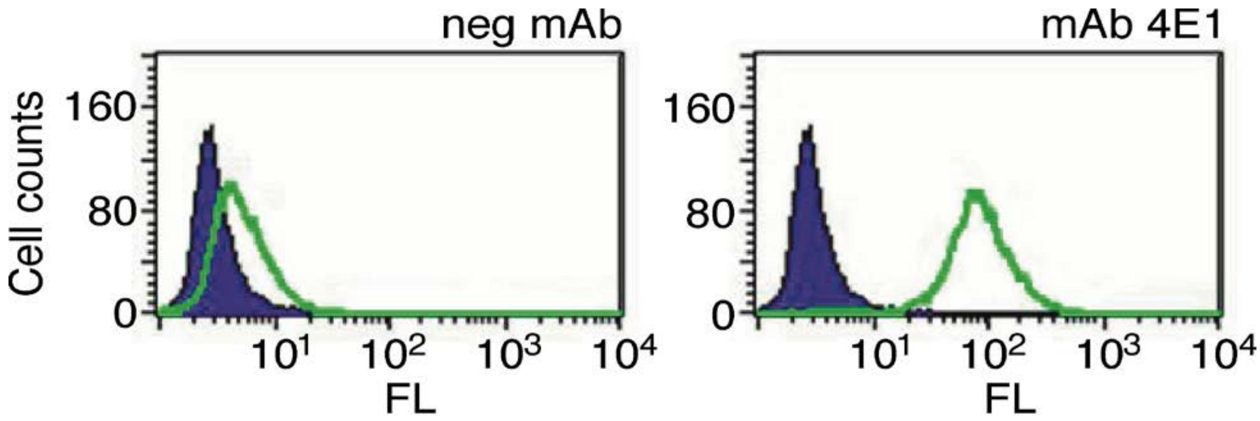

Supp figure 1: Flow cytometry analysis shows that 4E1 interacts with an endothelial cell surface antigen while an unrelated mAb (neg) does not. HUVEC grown in complete medium were detached from culture plates by EDTA treatment, resuspended as single cells in PBS and incubated with purified antibodies. The plots show cell fluorescence before (violet) and after (green) antibody binding.

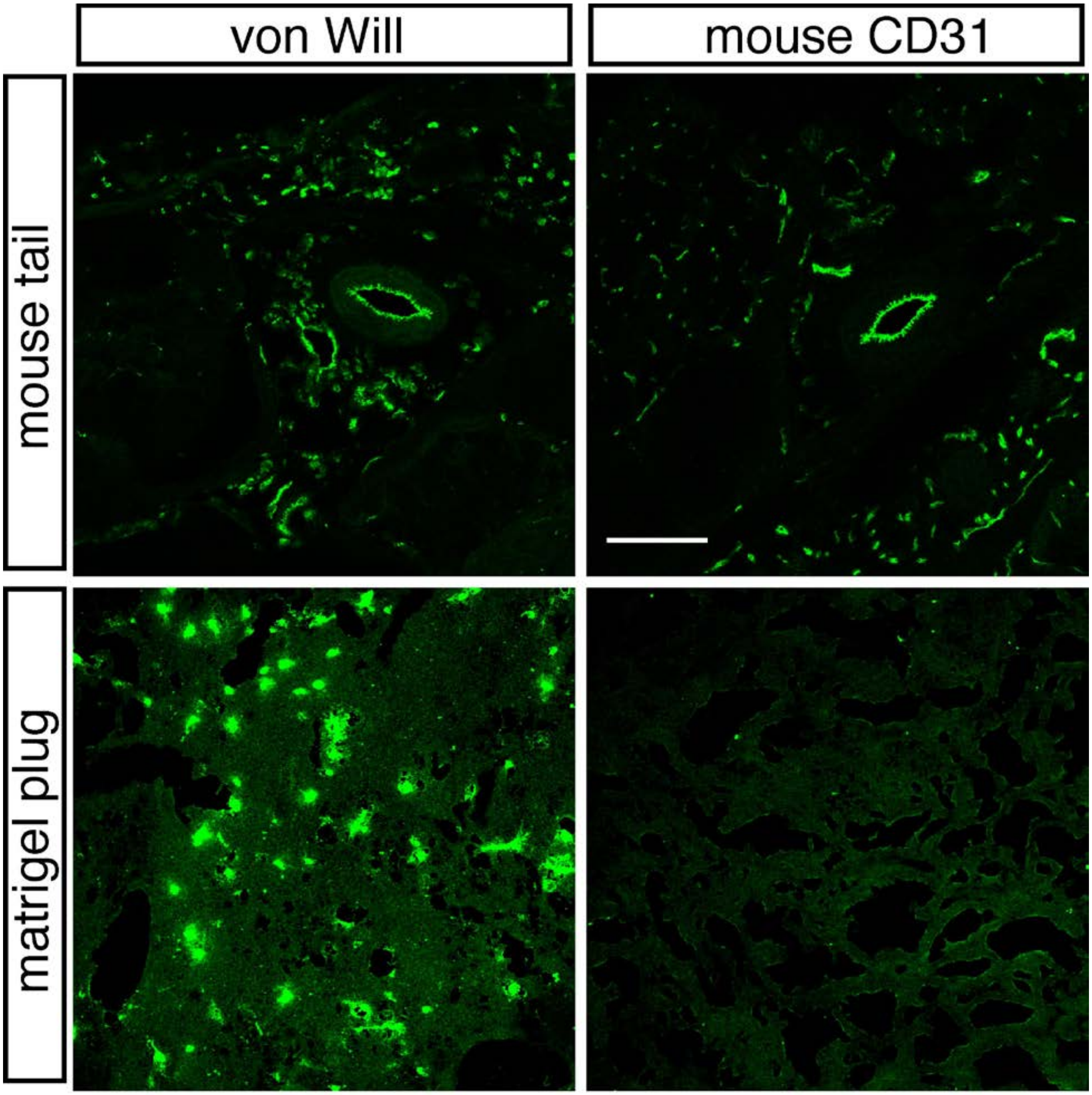

Supp figure 2: Serial sections of vessels through mouse tail and Matrigel plugs were stained by immunofluorescence using anti-CD31 and anti-von Willebrand factor antibodies. While the anti-von Willebrand factor antibodies cross-react with the protein of mouse and human origin, the anti-CD31 antibodies recognize specifically the mouse protein. Scale bar, 150 µm.

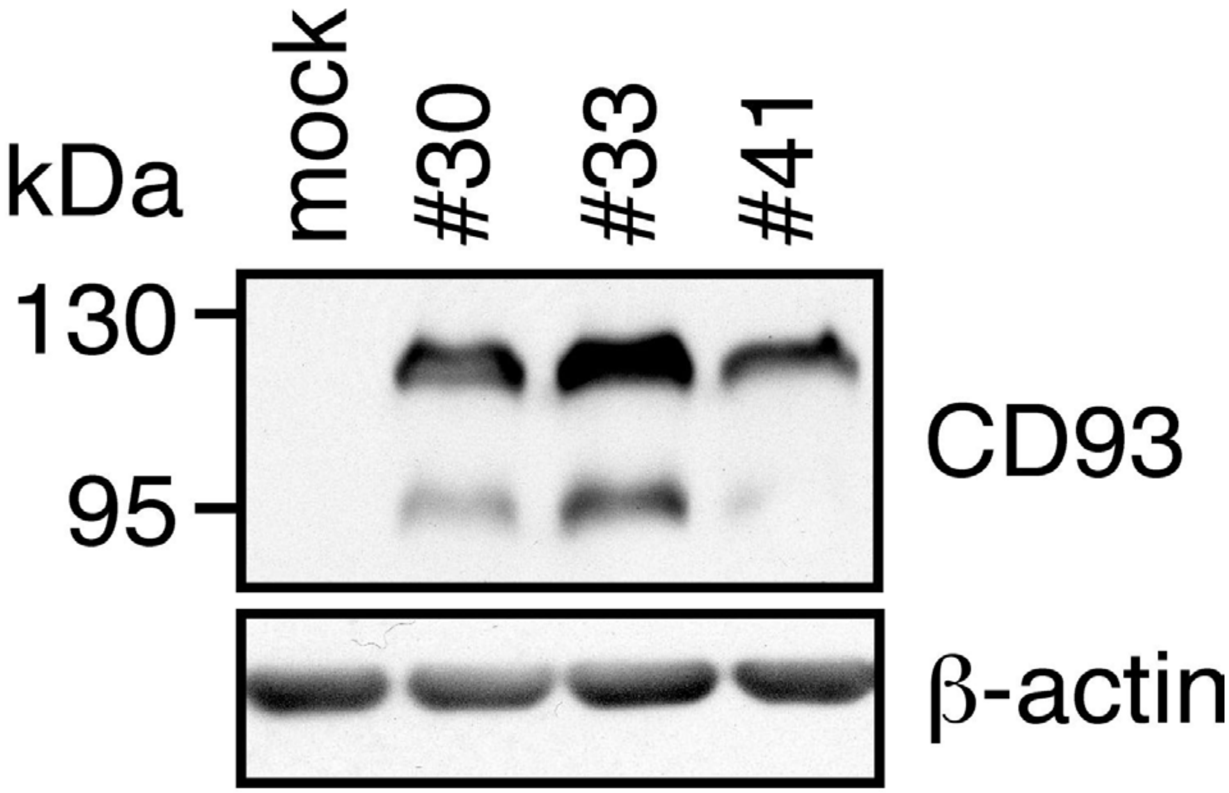

Supp figure 3:BALB/c fibroblasts were transiently transfected with empty vector (mock) or three different constructs expressing human CD93 (cDNA clones #30, #33, and #41). Total cell extracts were analyzed by Western blotting using anti-CD93 (H190) and anti-β-actin antibodies to confirm equal loading.

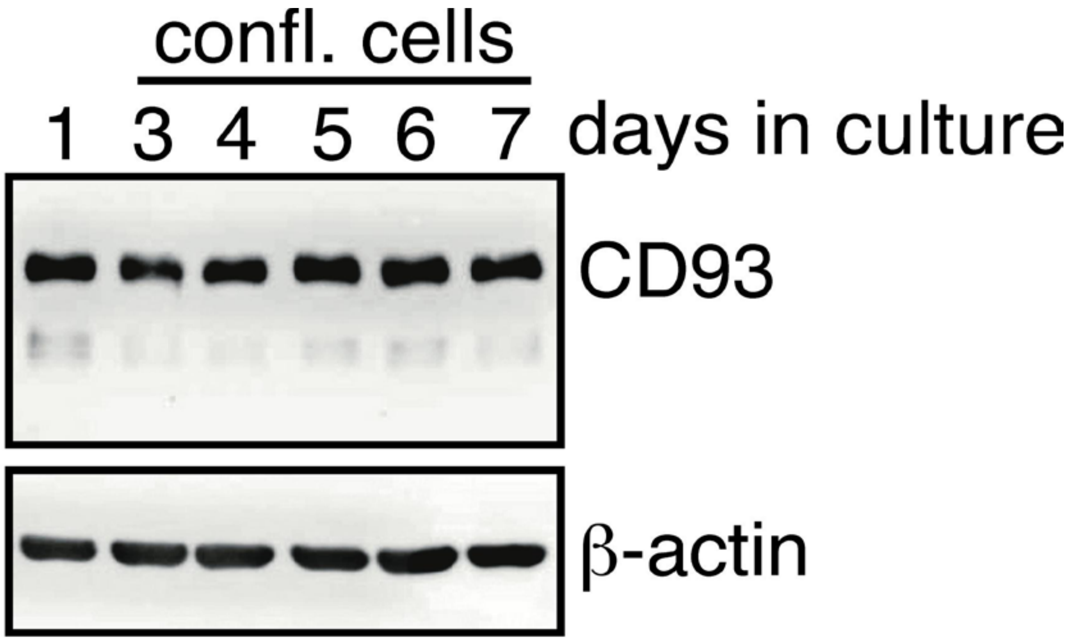

Supp figure 4:HUVEC were plated at low density and let to grow. Cell growth arrest occurred around day 3 when cells were fully confluent. At the times indicated cell extracts were collected and subjected to Western blot analysis by using anti-CD93 (H190) and anti-β-actin antibodies to confirm equal loading.

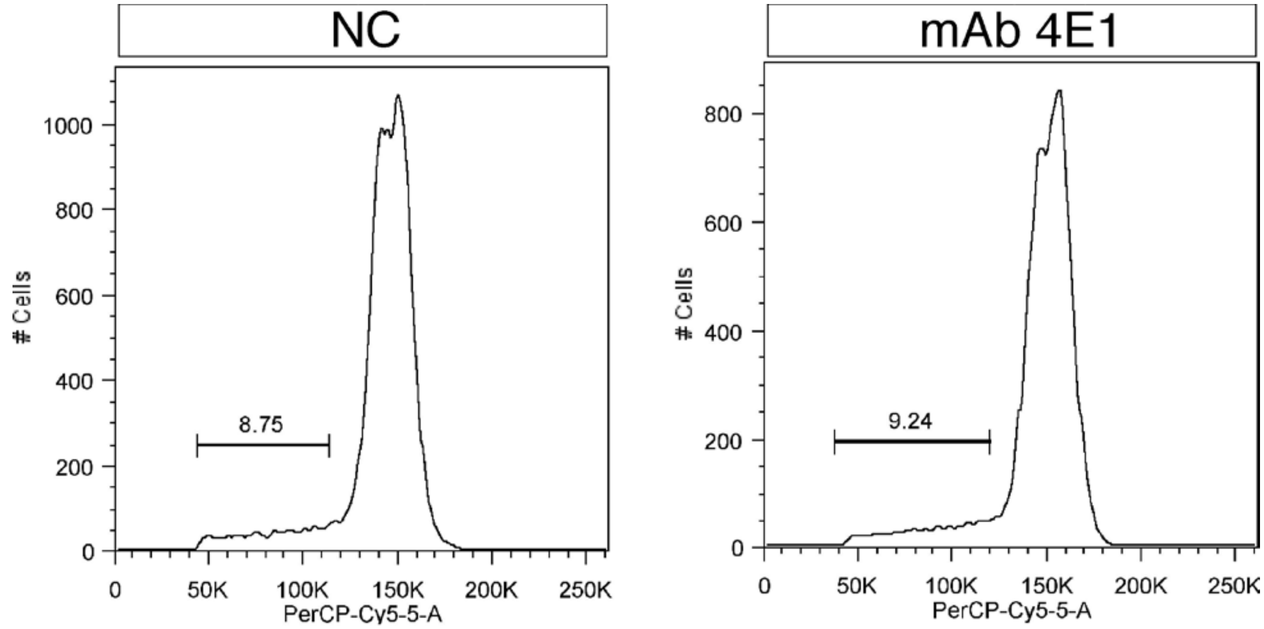

Supp figure 5: Representative flow cytometric analysis of nuclei of quiescent endothelial cells treated as in Fig. 3C. During the antibody treatment, cells were growth-arrested in G1 phase as confirmed by the absence of nuclei with duplicated DNA.

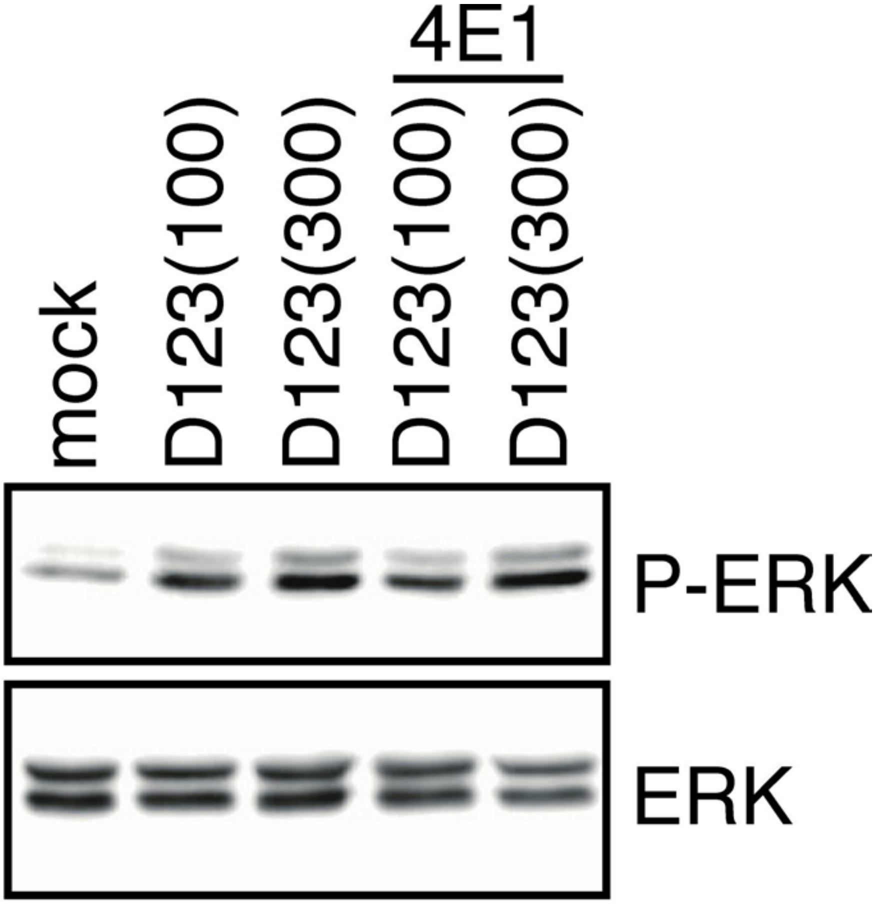

Supp figure 6: To prepare conditioned media containing recombinant proteins for the induction of phosphorylation in endothelial cells, Lenti-X 293T cells were transiently transfected with empty vector (mock) or a construct expressing CD93D123 mutant protein. 48 h after transfection cells were washed with PBS and grown in serum-free medium for 9 h. The harvested culture media were used as conditioned media and checked for the expression of soluble CD93D123 protein by immunoblotting with anti-Myc antibodies. HUVEC were serum-starved as previously described [15] and stimulated with 100 or 300 µl of conditioned medium. 300 µl of conditioned medium from mock transfected cells were used as negative control. 10 min after stimulation cells were lysated and the levels of phospho-ERK1/2 and total ERK1/2 were analyzed by immunoblotting with specific antibodies. In inhibition phosphorylation analysis, the mAb 4E1 was used at a concentration roughly thirtyfold higher than soluble CD93D123 protein, and before cell stimulation, conditioned medium was pretreated for 30 min with 4E1.
